# Comorbidities in HIV infected patients admitted to County Infectious Diseases Hospital Tg-Mureş in 2013

**DOI:** 10.1186/1471-2334-14-S4-P28

**Published:** 2014-05-29

**Authors:** Erzsébet Iringó Zaharia-Kézdi, Lucia Carmen Chiriac, Andrea Incze, Franciska Kristaly, Nina-Ioana Șincu

**Affiliations:** 1 University of Medicine and Pharmacy, Tîrgu Mureş, Romania

## 

The prevalence and incidence of comorbidities in HIV-infected patients has varied over the past 30 years, depending on the studied population. The purpose of this paper is to review the various conditions registered in HIV-positive patients admitted to the 1st Infectious Diseases Clinic of Tg-Mureş.

We performed a retrospective cross-sectional study, on 124 HIV infected patients (average age 27 years, 49 female, 85 subjects in AIDS stage), admitted to the HIV Department of the 1st Infectious Diseases Clinic of Tg-Mureş during January – December 2013 (253 hospitalizations, overall 2,415 days, median hospital stay 7 days). Co-infections (viral hepatitis, tuberculosis, bacterial, fungal, parasitic and other viral conditions), as well as pluri-organic comorbidities were noted and correlated to gender, level of immune-deficiency, history of HIV infection, adherence to antiretroviral therapy and outcome. Statistical analysis was performed with Mann-Whitney non parametric test.

Patients had an average of 6 comorbidities such as: 98 various bacterial, viral, fungal and parasitic infections, 91 hematologic disorders, 83 metabolic disorders, 77 gastrointestinal conditions, 68 acute respiratory disease cases, 43 neurological issues, 39 mental health problems, 35 hepatitis B infection, 32 chronic respiratory diseases, 25 cases of tuberculosis, 24 bone disorders, 12 cardiovascular diseases, 5 sexually transmitted diseases, and 2 malignancies. We registered statistically significant differences regarding the presence of comorbid conditions and gender (male>female, p=0.003), immune status (more comorbidities in patients with LT CD4<200/µL p=0.011), the time since initial HIV diagnosis (more comorbidities in older infections, p=0.03), level of adherence (more comorbidities in patients with low adherence, p=0.038) and outcome (more comorbidities in patients with bad outcome, death, p=0.01). Out of the 12 deaths, 10 were diagnosed with tuberculosis.

Despite their young age, our HIV-infected patients are suffering from a large number of comorbidities, so we can consider them cases of multimorbidity, leading to prolonged hospitalizations, therapeutic challenges and subsequently increased medical expenses.

